# BRIDGING SILOS: EVALUATION OF AN INNOVATIVE REGIONAL SENIOR TRANSPORTATION SERVICE

**DOI:** 10.1093/geroni/igac059.705

**Published:** 2022-12-20

**Authors:** Taylor Jansen, Nina Silverstein, Beth Dugan, Chae Man Lee, Shu Xu, YanJhu Su

**Affiliations:** University of Massachusetts Boston, Boston, Massachusetts, United States; University of Massachusetts Boston, Boston, Massachusetts, United States; University of Massachusetts Boston, Boston, Massachusetts, United States; University of Massachusetts Boston, Boston, Massachusetts, United States; University of Massachusetts Boston, Boston, Massachusetts, United States; University of Massachusetts Boston, Boston, Massachusetts, United States

## Abstract

A pilot study was conducted for a regional transit authority (RTA) to evaluate the implementation of a customized software to increase ridership, trips, destinations and coordination of rides among four Councils on Aging (COA) using RTA vans to serve rural communities in north central Massachusetts. Baseline and follow up ride data were collected from each COA from Fall 2019 (N= 178 riders; N= 4,230 trips) and Fall 2021 (N= 131 riders; N= 2,020 trips) and from 59 stakeholder interviews with riders, drivers, dispatchers, and staff from the COAs, RTA, and software company. The evaluation found after 6 months that while the goals of the pilot were not yet achieved, due in part to external factors such as the COVID-19 pandemic and driver shortage, valuable lessons were learned including the importance of balancing provider goals of efficiency and reporting with rider needs of driver hands-on assistance and friendly scheduling support.

